# Investigation of Endoscopic and Pathologic Features for Safe Endoscopic Treatment of Superficial Spreading Early Gastric Cancer: Erratum

**DOI:** 10.1097/01.md.0000484985.35690.8d

**Published:** 2016-06-24

In the article “Investigation of Endoscopic and Pathologic Features for Safe Endoscopic Treatment of Superficial Spreading Early Gastric Cancer”,^1^ which appeared in Volume 95, Issue 14 of *Medicine*, the affiliations section was formatted incorrectly. The article has since been corrected online.

## References

[1] Lee KJ Pak KH Hyung WJ Investigation of endoscopic and pathologic features for safe endoscopic treatment of superficial spreading early gastric cancer. *Medicine.* 2016 95:e3242. 27057862 10.1097/MD.0000000000003242 PMC4998778

